# Viral to metazoan marine plankton nucleotide sequences from the *Tara* Oceans expedition

**DOI:** 10.1038/sdata.2017.93

**Published:** 2017-08-01

**Authors:** Adriana Alberti, Julie Poulain, Stefan Engelen, Karine Labadie, Sarah Romac, Isabel Ferrera, Guillaume Albini, Jean-Marc Aury, Caroline Belser, Alexis Bertrand, Corinne Cruaud, Corinne Da Silva, Carole Dossat, Frédérick Gavory, Shahinaz Gas, Julie Guy, Maud Haquelle, E'krame Jacoby, Olivier Jaillon, Arnaud Lemainque, Eric Pelletier, Gaëlle Samson, Mark Wessner, Genoscope Technical Team, Pascal Bazire, Odette Beluche, Laurie Bertrand, Marielle Besnard-Gonnet, Isabelle Bordelais, Magali Boutard, Maria Dubois, Corinne Dumont, Evelyne Ettedgui, Patricia Fernandez, Espérance Garcia, Nathalie Giordanenco Aiach, Thomas Guerin, Chadia Hamon, Elodie Brun, Sandrine Lebled, Patricia Lenoble, Claudine Louesse, Eric Mahieu, Barbara Mairey, Nathalie Martins, Catherine Megret, Claire Milani, Jacqueline Muanga, Céline Orvain, Emilie Payen, Peggy Perroud, Emmanuelle Petit, Dominique Robert, Murielle Ronsin, Benoit Vacherie, Silvia G. Acinas, Marta Royo-Llonch, Francisco M. Cornejo-Castillo, Ramiro Logares, Beatriz Fernández-Gómez, Chris Bowler, Guy Cochrane, Clara Amid, Petra Ten Hoopen, Colomban De Vargas, Nigel Grimsley, Elodie Desgranges, Stefanie Kandels-Lewis, Hiroyuki Ogata, Nicole Poulton, Michael E. Sieracki, Ramunas Stepanauskas, Matthew B. Sullivan, Jennifer R. Brum, Melissa B. Duhaime, Bonnie T. Poulos, Bonnie L. Hurwitz, Tara Oceans Consortium Coordinators, Silvia G. Acinas, Peer Bork, Emmanuel Boss, Chris Bowler, Colomban De Vargas, Michael Follows, Gabriel Gorsky, Nigel Grimsley, Pascal Hingamp, Daniele Iudicone, Olivier Jaillon, Stefanie Kandels-Lewis, Lee Karp-Boss, Eric Karsenti, Fabrice Not, Hiroyuki Ogata, Stéphane Pesant, Jeroen Raes, Christian Sardet, Michael E. Sieracki, Sabrina Speich, Lars Stemmann, Matthew B. Sullivan, Shinichi Sunagawa, Patrick Wincker, Stéphane Pesant, Eric Karsenti, Patrick Wincker

**Affiliations:** 1 CEA—Institut de Biologie François Jacob, Genoscope, 2 rue Gaston Crémieux, Evry 91057, France; 2 CNRS, UMR 7144, Station Biologique de Roscoff, Place Georges Teissier, Roscoff 29680, France; 3 Sorbonne Universités, UPMC Univ Paris 06, UMR 7144, Station Biologique de Roscoff, Place Georges Teissier, Roscoff 29680, France; 4 Departament de Biologia Marina i Oceanografia, Institute of Marine Sciences (ICM), CSIC, Barcelona E08003, Spain; 5 CNRS, UMR 8030, Evry CP5706, France; 6 Université d'Evry, UMR 8030, Evry CP5706, France; 7 FONDAP Center for Genome Regulation, Moneda 1375, Santiago 8320000, Chile; 8 Laboratorio de Bioinformática y Expresión Génica, Instituto de Nutrición y Tecnología de los Alimentos (INTA), Universidad de Chile, El Libano Macul, Santiago 5524, Chile; 9 Ecole Normale Supérieure, PSL Research University, Institut de Biologie de l’Ecole Normale Supérieure (IBENS), CNRS UMR 8197, INSERM U1024, 46 rue d’Ulm, Paris F-75005, France; 10 European Molecular Biology Laboratory, European Bioinformatics Institute, Wellcome Genomes Campus, Hinxton, Cambridge CB10 1 SD, UK; 11 CNRS UMR 7232, BIOM, Avenue Pierre Fabre, Banyuls-sur-Mer 66650, France; 12 Sorbonne Universités Paris 06, OOB UPMC, Avenue Pierre Fabre, Banyuls-sur-Mer 66650, France; 13 Directors’ Research European Molecular Biology Laboratory, Meyerhofstr. 1, Heidelberg 69117, Germany; 14 Structural and Computational Biology, European Molecular Biology Laboratory, Meyerhofstr. 1, Heidelberg 69117, Germany; 15 Institute for Chemical Research, Kyoto University, Gokasho, Uji, Kyoto 611-0011, Japan; 16 Bigelow Laboratory for Ocean Sciences, East Boothbay, Maine 04544, USA; 17 National Science Foundation, Arlington, Virginia 22230, USA; 18 Departments of Microbiology and Civil, Environmental and Geodetic Engineering, Ohio State University, Columbus, Ohio 43210, USA; 19 Department of Microbiology, The Ohio State University, Columbus, Ohio 43210, USA; 20 Department of Ecology and Evolutionary Biology, University of Michigan, Ann Arbor, Michigan 48109, USA; 21 University of Arizona, Tucson, Arizona 85721, USA; 22 Department of Agricultural and Biosystems Engineering, University of Arizona, Tucson, Arizona 85719, USA; 23 MARUM, Center for Marine Environmental Sciences, University of Bremen, Leobener Str. 8, Bremen 28359, Germany; 24 PANGAEA, Data Publisher for Earth and Environmental Science, University of Bremen, Leobener Str. 8, Bremen 28359, Germany; 25 Sorbonne Universités, UPMC Université Paris 06, CNRS, Laboratoire d’oceanographie de Villefranche (LOV), Observatoire Océanologique, 181 Chemin du Lazaret, Villefranche-sur-mer F-06230, France; 26 Molecular Medicine Partnership Unit, University of Heidelberg and European Molecular Biology Laboratory, Heidelberg 69120, Germany; 27 Max Delbrück Centre for Molecular Medicine, Berlin 13125, Germany; 28 Department of Bioinformatics, University of Wuerzburg, Würzburg 97074, Germany; 29 School of Marine Sciences, University of Maine, Orono, Maine 04469, USA; 30 Department of Earth, Atmospheric and Planetary Sciences, Massachusetts Institute of Technology, Cambridge, Massachusetts, USA; 31 Aix Marseille Univ, Université de Toulon, CNRS, IRD, MIO, Marseille, France; 32 Stazione Zoologica Anton Dohrn, Villa Comunale, Naples 80121, Italy; 33 Department of Microbiology and Immunology, Rega Institute, KU Leuven, Herestraat 49, Leuven 3000, Belgium; 34 VIB Center for Microbiology, Herestraat 49, Leuven 3000, Belgium; 35 CNRS, UMR 7009 Biodev, Observatoire Océanologique, Villefranche-sur-mer F-06230, France; 36 Laboratoire de Physique des Océans, UBO-IUEM, Place Copernic, Plouzané 29820, France; 37 Department of Geosciences, Laboratoire de Météorologie Dynamique (LMD), Ecole Normale Supérieure, 24 rue Lhomond, Paris, Cedex 05 75231, France; 38 Institute of Microbiology, Department of Biology, Vladimir-Prelog-Weg 4, Zürich 8093, Switzerland.

**Keywords:** Water microbiology, Molecular biology, Metagenomics

## Abstract

A unique collection of oceanic samples was gathered by the *Tara* Oceans expeditions (2009–2013), targeting plankton organisms ranging from viruses to metazoans, and providing rich environmental context measurements. Thanks to recent advances in the field of genomics, extensive sequencing has been performed for a deep genomic analysis of this huge collection of samples. A strategy based on different approaches, such as metabarcoding, metagenomics, single-cell genomics and metatranscriptomics, has been chosen for analysis of size-fractionated plankton communities. Here, we provide detailed procedures applied for genomic data generation, from nucleic acids extraction to sequence production, and we describe registries of genomics datasets available at the European Nucleotide Archive (ENA, www.ebi.ac.uk/ena). The association of these metadata to the experimental procedures applied for their generation will help the scientific community to access these data and facilitate their analysis. This paper complements other efforts to provide a full description of experiments and open science resources generated from the *Tara* Oceans project, further extending their value for the study of the world’s planktonic ecosystems.

## Background & Summary

Systems-level studies of the functional biodiversity of marine ecosystems are becoming crucial for understanding and managing ocean resources. During the *Tara* Oceans expeditions (2009–2013), original and innovative strategies were used to gather the largest modern-day collection of marine plankton in combination with an extensive suite of environmental data^1^. The worldwide sampling strategy and methodology are presented in Pesant *et al.*^2^.

Here we focus on the description of procedures applied for genetic analysis of samples collected during the *Tara* Oceans campaigns. During the last decade, rapid advances in sequencing technology has been a major force in the rise of studies aimed at deciphering genetic and functional biodiversity in complex environmental samples^3,4^. Global metagenomic approaches have been shown to be successful in providing extensive information about organism abundance and gene content within a sample^5–9^, whereas metatranscriptomics, based on massive sequencing of microbial community cDNA, has emerged as a powerful tool for revealing functional genes and metabolic pathways in diverse environments such as marine^10–12^, soil^13–15^ or human internal organs^16^ ecosystems.

**Figure 1:**
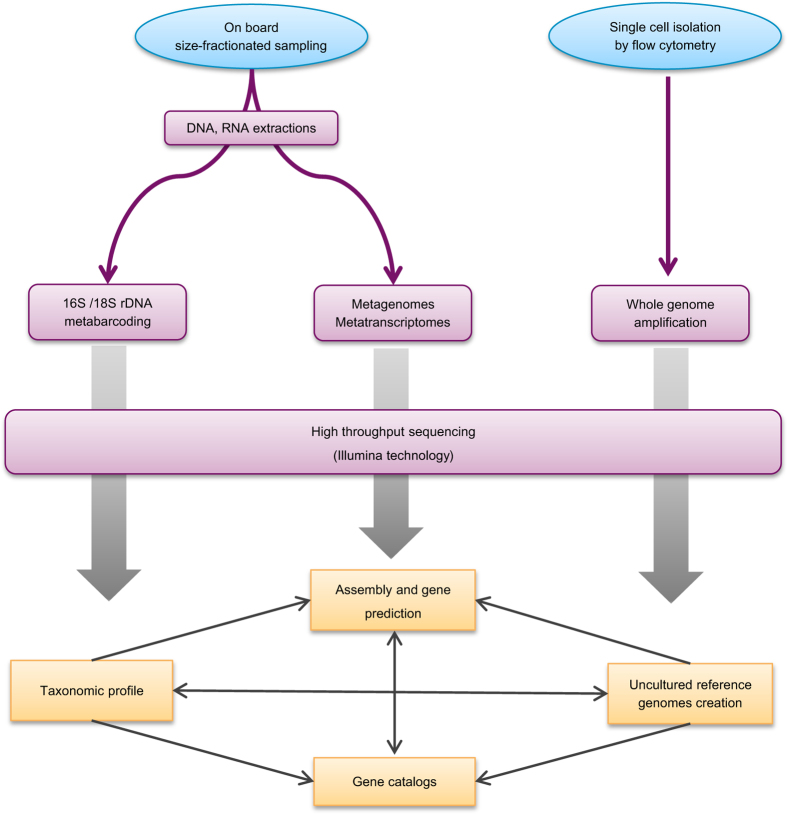
Overview of -omics analysis strategy applied on *Tara* Oceans samples.

More than 1,600 environmental samples collected during the *Tara* Oceans expedition were processed for -omics analyses. The particular sampling strategy, based on size fractionation, allowed recovery of five groups of organisms: viruses, giant viruses (giruses), prokaryotes (bacteria and archaea), unicellular eukaryotes (protists) and metazoans (see ref. 2 for a detailed description). In order to enhance the value of this unique sample collection and to unveil the structure and function of plankton communities, we adopted a sequencing strategy that relies on metabarcoding, metagenomics, single-cell genomics and metatranscriptomics approaches (Fig. 1 and Table 1).

Samples from all size fractions underwent DNA extraction and sequencing library preparation for metagenomics analyses. Purified DNA from eukaryotic and prokaryotic-enriched fractions was also used for generation of phylogenetic tags from 18S and 16S rRNA genes in order to help defining the taxonomic composition of each sampling site. In parallel, the same eukaryotic and prokaryotic-enriched fractions were used to produce metatranscriptomes by converting extracted RNA into cDNA. Because marine eukaryotes are only poorly represented in databases^17^, further efforts were made to produce reference genomes for some uncultured unicellular organisms. This was possible through single cell isolation by flow cytometry followed by whole genome amplification and *de novo* sequencing.

A first wave of analyses performed using a subset of *Tara* Oceans samples generated high quality data leading to several publications^18–29^ that shed light on the extraordinary biodiversity of marine ecosystems. Among the most significant results, metagenomic data obtained from prokaryote- and virus-enriched samples were used to establish an ocean microbial reference catalog with more than 40 million genes^23^. In parallel, viral metagenomes combined with morphological datasets were used to assess viral community patterns and structure, providing a map of global dsDNA viral diversity in the surface- and deep ocean^20,29^ whereas for protists and metazoans, 18S rRNA metabarcoding allowed to profile eukaryotic diversity in the photic zone^21^. However, the utility of *Tara* Oceans metadata have been only partially explored until now and further publications will help disentangling one of the largest and most complex ecosystems on Earth.

This paper aims to gather together all procedures used to generate sequencing data, from nucleic acids (DNA/RNA) extraction to sequence production. Experimental methods, mostly already published in *Tara* Oceans related papers, are here described extensively, adding detailed information about quality control processes applied to each experimental step and to generated datasets. Most importantly, the associated Metadata Record allows straightforward linking of the described methods with genomics datasets already available openly. This paper is an important contribution to better assess already published work, and to support further analyses of genomics data generated by the *Tara* Oceans expedition.

## Methods

The generation of information-rich data from marine plankton samples presents unique challenges that are inherent to the particular sampling conditions at sea and the wide spectrum of organisms included in that environment. All processing steps, including biomass collection, sample preservation, nucleic acids extractions and sequencing library preparation, are critical and require specific protocols and robust methods in order to ensure comparability of results and limit potential biases^30–32^.

Our methods were either developed specifically for *Tara* Oceans samples or carefully selected among existing ones in order to meet the requirements of our sequencing strategy and to produce optimized datasets for downstream bioinformatics analyses, as for example the production of overlapping reads from metagenomics libraries to facilitate assembly. They are presented in five sub-sections, starting with a brief description of how samples were handled between the research vessel and the processing laboratories (Subsection 1). Sub-section 2 reports on DNA and RNA extractions procedures for -omics analyses, including the generation of amplified genomic DNA from uncultured isolated unicellular eukaryotes. The generation of 18S and 16S rRNA amplicons from DNA of specific size-fractions is described in subsection 3 and Illumina libraries preparation in subsection 4. Sequencing procedures and post-sequencing data processing are described in subsection 5. For details on the onboard sampling protocols, see Pesant *et al.*^2^.

### [1] Handling of genomics samples

Genomics samples were transferred on average every 6 weeks from a port of call to the European Molecular Biology Laboratory (EMBL, Heidelberg) in Germany. Transportation was organized by experts from World Courier (www.worldcourier.com) who ensured that the chain of cold was never broken. At EMBL, samples were sorted, repackaged according to their final destination, and transported again by World Courier to the different laboratories responsible for their analysis (Table 1).

In the respective laboratories, samples were immediately identified by scanning/reading their barcode label and were stored in cryo boxes or in −80 °C freezers. During all these steps, samples were manipulated on dry ice. Each laboratory used its own sample management system to record the storage location and to monitor sample usage.

### [2] Nucleic acids preparations

Different nucleic acids extraction methods were applied to obtain DNA and RNA from the different plankton groups sampled during *Tara* Oceans Expedition.

*2.1. DNA/RNA extractions from size fractions 0.8–5 μm (or 0.8–3 μm), 0.8–2000 μm, 5–20 μm (or 3–20 μm), 3–2000 μm, 20–180 μm and 180–2000 μm (Method ID: Euk_ DNA_RNA_ext)*

Plankton from these size fractions was collected on membrane filters and targeted unicellular eukaryotes (protists), usually<200 μm, and metazoans, usually >200 μm.

The protocol applied for nucleic acid extractions was based on simultaneous extraction of DNA and RNA by cryogenic grinding of cryopreserved membrane filters followed by nucleic acid extraction with NucleoSpin RNA kits (Macherey-Nagel, Düren, Germany) combined with DNA Elution buffer kit (Macherey-Nagel). This protocol was derived from optimization and validation experiments in the de Vargas laboratory at the Station Biologique de Roscoff (France). In particular, this preliminary work aimed principally to adapt the efficiency of the cell disruption and DNA/RNA extraction steps in order to efficiently capture nucleic acids from protists and metazoans collected from sea water filtering. Tests were conducted on a mock community composed of 26 monoclonal strains from the Roscoff Culture Collection (http://roscoff-culture-collection.org/) and natural filter samples collected in Roscoff (ASTAN, SOMLIT sampling). During these tests, the cell disruption step was optimized by applying a mechanical cryogrinding method to be sure that cells were efficiently disrupted, minimizing RNA and DNA degradation. Three cryogrinding protocols were tested using a 6,770 Freezer/Mill or 6,870 Freezer/Mill instrument (SPEX SamplePrep, Metuchen, NJ): 1 grinding cycle at 5 knocks per second for 1 min, 1 grinding cycle at 10 knocks per second for 1 min, and 2 grinding cycles at 10 knocks per second for 1 min. Best RNA and DNA quantities were obtained using 2 grinding cycles at 10 knocks per second for 1 min. Then, three simultaneous DNA /RNA extraction protocols were compared: Trizol method followed by RNeasy purification kit (Qiagen, Hilden, Germany), NucleoSpin RNA kit combined with DNA elution buffer set (Macherey-Nagel), and Nucleobond kit (Macherey-Nagel). Best quality results (DNA and RNA integrity conservation, ratios A260/280 and A260/230) were obtained with NucleoSpin RNA kit combined with DNA elution buffer set.

After validation, the procedure described herein was applied in the Genoscope laboratory on *Tara* Oceans filters from the size fractions cited above.

Briefly, each membrane was accommodated into a grinding vial with 1 ml RA1 lysis buffer (Macherey-Nagel) and 1% β-mercaptoethanol (Sigma, St Louis, MO) and subjected to the following grinding program: 2 min pre-cooling time, first grinding cycle at 10 knocks per second for 1 min, 1 min cooling time and final grinding cycle at 10 knocks per second for 1 min. Cryogrinded powder was resuspended in 2 ml RA1 lysis buffer with 1% β-mercaptoethanol, transferred to a large capacity NucleoSpin Filter from RNA Midi kit and centrifuged for 10 min at 1,500 *g*. After further addition of 1 ml RA1 lysis buffer with 1% β-mercaptoethanol, the filter was recentrifuged 3 min at 1,500 *g*. The eluate was transferred to a new tube with addition of 1 volume of ethanol 70%. The mixture was loaded to a NucleoSpin RNA Mini spin column and washed twice with DNA wash solution. DNA was eluted by three successive elutions each with 100 μl DNA elution buffer and stored in sterile microtubes at −20 °C. DNA was quantified by a dsDNA-specific fluorimetric quantitation method using Qubit 2.0 Fluorometer instrument with Qubit dsDNA BR (Broad range) and HS (High sensitivity) Assays (ThermoFisher Scientific, Waltham, MA). DNA quality was checked in a sample subset by running 1 μl on 0.7% agarose gel for 60 min at 100 V.

RNA purification was continued on the previous NucleoSpin RNA Mini spin column by digesting residual DNA with 10 μl rDNase and 90 μl reaction buffer for rDNase. After 15 min incubation at room temperature, the column was washed with RA2 and RA3 buffers. RNA was eluted in 60 μl RNase-free water and stored in sterile microtubes at −80 °C. Quantity and quality of extracted RNA were assessed with RNA-specific fluorimetric quantitation on a Qubit 2.0 Fluorometer using Qubit RNA HS Assay. The qualities of total RNA were checked by capillary electrophoresis on an Agilent Bioanalyzer, using the RNA 6,000 Pico LabChip kit (Agilent Technologies, Santa Clara, CA).

Finally, the RNA extraction procedure integrated an in-column DNase treatment but, based on previous experience with this method, this step was sometimes only partially effective and did not always preclude the presence of trace DNA in final RNA samples. DNA removal in RNA samples is essential to prevent the incorporation of any genomic material in the RNA-Seq library and consequently the misinterpretation of RNA-Seq data analyses. In order to reduce as far as possible the risk of residual genomic DNA, a further DNase treatment was applied as a precaution on total RNA extracted from samples collected in the second of the *Tara* Oceans expeditions (Polar Circle campaign, stations 155–210). After extraction and quality control assessment as described above, these RNA samples were further processed as follows: a quantity of 5 μg, or less, total RNA aliquots were treated with Turbo DNA-free kit (Thermo Fisher Scientific), according to the manufacturer’s DNase treatment protocol. After two rounds of 30 min incubation at 37 °C, the reaction mixture was purified with the RNA Clean and Concentrator-5 kit (ZymoResearch, Irvine, CA) following the procedure described for retention of >17 nt RNA fragments. RNA was eluted in 9–15 μl nuclease-free water by two elution steps in order to maximize recovery. Purified RNA was quantified with Qubit RNA HS Assay. Initially, the efficiency of DNase treatment was assessed by PCR, showing that the treatment was efficient in all checked samples. Due to the large number of samples to be treated, this validation step was then omitted.

*2.2. DNA/RNA extractions from size fractions 0.2–1.6 μm and 0.2–3 μm*

Two different protocols were applied to these size fractions that mainly targeted prokaryotes. Viruses and giant viruses (giruses) were also recovered in these fractions although dedicated filters (e.g.,<0.22 μm) and specific extractions protocols (described in Sections 2.4 and 2.5) were allocated for their analysis.

### Acinas lab DNA extraction (Method ID: Acinas_prok_DNA_extr)

Half of the 0.22 μm 142-mm-diameter Millipore polyethersulfone Express Plus membrane filter (Merck Millipore, Billerica, MA) was cut into small pieces and soaked in 3 ml lysis buffer (40 mM EDTA, 50 mM Tris-HCl, 0.75 M sucrose). Lysozyme (1 mg ml^−1^ final concentration) was added and samples were incubated at 37 °C for 45 min while slightly shaken. Then, sodium dodecyl sulfate (1% final concentration) and proteinase *K* (0.2 mg ml^−1^ final concentration) were added and samples were incubated at 55 °C for 60 min while slightly shaken. The lysate was collected and processed with the standard phenol-chloroform extraction procedure: an equal volume of Phenol:Chloroform:Isoamyl Alcohol (25:24:1, v/v) was added to the lysate, carefully mixed and centrifuged 10 min at 3,000 *g*. Then the aqueous phase was recovered and the procedure was repeated once. Finally, an equal volume of Chloroform:Isoamyl Alcohol (24:1, v/v) was added to the recovered aqueous phase in order to remove residual phenol. The mixture was centrifuged and the aqueous phase was recovered for further purification. The aqueous phase was then concentrated and purified by centrifugation with a Centricon concentrator (Amicon Ultra-4 Centrifugal Filter Unit with Ultracel-100 membrane, Millipore). Once the aqueous phase was concentrated, this step was repeated three times adding 2 ml of sterile MilliQ water each time in order to purify the DNA. After the third washing step, between 100 and 250 μl of purified total genomic DNA product was recovered per sample. Isolated DNA was quantified using a Nanodrop ND-1000 spectrophotometer (NanoDrop Technologies Inc, Wilmington, DE, USA) and its integrity was checked on an agarose gel (1.5%).

### Acinas lab RNA extraction (Method ID: Acinas_prok_RNA_ext)

RNA isolation was performed using the RNeasy Mini kit (Qiagen) with a modified protocol. The filters were cut into small pieces and washed with pre chilled PBS buffer in order to eliminate the RNA Later. To avoid loss of any cell during the washing process, the washing solution was passed through a glass cup filtration system including a filter of 0.22 μm pore-size. The pieces of the filter and the extra filter were placed in a 50 ml falcon tube with a mixture of beads (1 ml of 0.5 mm glass beads and 2 ml of 0.1 mm Zirconia beads (BioSpec Products)) and 10 ml buffer RLT+1% β-mercaptoethanol. The mixture was shaken during 15 min in a MoBio Vortex (Vortex-Genie 2, MO BIO Laboratories, Inc.) and then centrifuged at 4,500 *g* 5 min at 4 °C. The supernatant was transferred to a new 50 ml falcon tube and centrifuged again for 10 min at 4,500 *g*. The supernatant (about 10 ml) was transferred to a new 50 ml tube, 1 volume 70% ethanol was added to the lysate and mixed by inversion four times. The mixture was divided in two and each volume loaded separately (by successive 700 μl aliquots) in two RNeasy mini columns and filtered using a vacuum pump. In this way, a better yield is obtained rather than putting the entire volume in a single column. Then, each column was washed with 700 μl Buffer RW1 and twice with 500 μl Buffer RPE according the manufacturer protocol. No DNase treatment was applied to the column. Finally, RNA was eluted from the membrane with 70 μl RNase-free water. The RNA was quantified using both a Nanodrop ND-1000 spectrophotometer (NanoDrop Technologies Inc, Wilmington, DE, USA) and Qubit Fluorometer. Extracted RNA samples were then sent to Genoscope where, prior to further processing, a DNase treatment using Turbo DNA-free kit was performed as described at the end of Section 2.1.

### Genoscope lab DNA/RNA extraction (Method ID: Genoscope_prok_RNA_ext)

An alternative protocol was developed in a second time and applied to the majority of samples. This procedure was based on cryogenic grinding followed by DNA/RNA purification with Nucleospin RNA kit as previously described for protists- and metazoans- enriched nucleic acids isolation. The following modifications were applied in order to efficiently grind larger and thicker 0.2–1.6 μm and 0.2–3 μm filters: the membranes were cut in many small pieces, dispatched equally in two vials and cryocrushed applying the same conditions previously described. Cryogrinded powders from each vial were resuspended in 2 ml RA1 lysis buffer in the presence of 1% β-mercaptoethanol, then pooled together and transferred to a single NucleoSpin Filter Midi. After a first centrifugation for 20 min at 1,500 *g*, 1 ml RA1 lysis buffer with 1% β-mercaptoethanol was added to the column which was then recentrifuged 3 min at 1,500 *g*. Then, extraction was continued following the same procedure applied to protists- and metazoans- enriched samples, including the additional post-extraction DNase treatment, already described in Section 2.1.

*2.3. DNA and RNA backups*

After nucleic acids extractions, two RNA aliquots and three DNA aliquots were prepared for each sample. One aliquot was used for the library preparation and sequencing process, the second one was stored as a backup. If RNA quantity was<100 ng, backup copy was omitted.

In the case of DNA, the third aliquot was used to produce an amplified DNA backup by whole genome amplification (WGA) by using Illustra GenomiPhi DNA Amplification Kit (GE Healthcare, Little Chalfont, UK). Briefly, 10 ng of DNA were diluted in 25 μl sample buffer and denatured for 3 min at 95 °C. After cooling on ice, samples were mixed to 22.5 μl reaction buffer containing random hexamers and 2.5 μl Phi29 enzyme mix and incubated at 30 °C for 3 hours. After amplification, Phi29 DNA polymerase was heat inactivated during 10 min at 65 °C. In order to reduce hyperbranched DNA regions generated by WGA process, amplified DNA was incubated with RepliPhi phi29 DNA polymerase (Epicentre Biotechnologies, Madison, WI) without any primer at 30 °C for 2 hours and 65 °C for 3 min, followed by S1 nuclease (Thermo Fisher Scientific) digestion at 37 °C for 30 min^33^. After DNA cleanup with Agencourt GenFind V2 System omitting the lysis step (Beckman Coulter Genomics, Danvers, MA), internal nicks were repaired by adding 100 U *E. coli* DNA polymerase I (New England Biolabs, Ipswich, MA) in 100 μl 1x NEB buffer 2 and 4 mM dNTP and incubating at 25 °C for 30 min. DNA was purified again with Agencourt GenFind V2 System and resuspended in 200 μl elution buffer. DNA was quantified with Qubit dsDNA BR and HS Assays and subjected to quality check by running 1 μl on 0.7% agarose gel for 60 min at 100 V.

*2.4. Viral particle concentration and DNA extractions from size fraction <0.22 μm (Method ID: virus_DNA_ext)*

This protocol describes a technique to recover viruses from natural waters using iron-based flocculation and large-pore-size filtration, followed by resuspension of virus-containing precipitates in a pH 6 buffer^34^.

Briefly, FeCl_3_ precipitation^34^ was used to concentrate viruses from 20–60 l of 0.22 μm filtered seawater, which were then resuspended in ascorbate buffer (0.125 M Tris-base, 0.1 M sodium EDTA dehydrate, 0.2 M magnesium chloride hexahydrate, 0.2 M ascorbate). This Fe-based virus flocculation, filtration and resuspension method (FFR) is efficient (>90% recovery), reliable, inexpensive and adaptable to many aspects of marine viral ecology and genomics research. Data are also available from replicated metagenomes to help researchers’ decisions on the impact of linker amplification methods from low input DNA^35^, viral purification strategies^36^, and library preparation and sequencing platform choices^36^. Following resuspension, recovered viruses were treated with DNase I to remove free DNA^37^, followed by the addition of 0.1 M EDTA and 0.1 M EGTA to halt DNase activity, and further concentrated to<1 ml using an Amicon 100 KDa filter (Sigma). DNA was extracted using the Wizard Prep DNA Purification system (Promega, Madison, WI). DNA concentration was assessed with PicoGreen (Thermo Fisher Scientific). All detailed protocols are listed by name and are documented and available at https://www.protocols.io/groups/sullivan-lab.

*2.5. DNA extractions from sizData availability e fractions 0.2–1.6 μm, 0.1–0.2 μm, 0.45–0.8 μm, 0.2–0.45 μm (Method ID: girus_DNA_ext)*

These size fractions were used to target giant viruses (giruses). DNA was extracted using a modified CTAB protocol^38,39^. Filters were crushed in liquid nitrogen, incubated at 60 °C for one hour in a CTAB buffer, DNA was purified using an equal volume of chloroform/isoamyl alcohol (24:1) and a one-hour-long-RNase digestion step. DNA was precipitated with a 2/3 volume of isopropanol and washed with 1 ml of a solution containing 76% v/v ethanol and 10 mM ammonium acetate solution. Finally, the extracted DNA samples were dissolved in 100 μl of laboratory grade deionized water and stored at −20 °C until the sequencing steps.

*2.6. Preparation of single cell amplified genomes (SAGs) (Method ID: SAGs_amplif)*

Single amplified genomes (SAGs) were generated and their taxonomic assignments were obtained as in Martinez-Garcia *et al.*^40^ with the following modifications. Samples for heterotrophic (aplastidic) cells were stained using SYBR Green I^41^. Samples for phototrophic (plasidic) cells were unstained. No attempt was made to identify mixotrophic cells. Several 384-well plates containing single cells of each type were prepared from each environmental sample. Backup plates were stored frozen at −80 °C. Single cell amplifications were validated by using an aliquot for PCR with eukaryotic universal 18S primers. SAGs with positive 18S sequence were sent to Genoscope for whole genome sequencing. Upon arrival, 2.5 μl were removed from each well and used to generate an amplified DNA backup by WGA. The reactions were performed as previously described for DNA extractions (section 2.3) except that debranching reactions were omitted and instead amplified DNA was purified by QIAamp DNA Mini kit (Qiagen).

### [3] 18S and 16S rRNA genes amplicon generation for eukaryotic and prokaryotic metabarcoding

To address general questions of eukaryotic biodiversity over extensive taxonomic and ecological scales, the hypervariable loop V9 of the 18S rRNA gene was targeted for amplicon generation using DNA extracted from eukaryote-enriched fractions (0.8–5 μm or 0.8–3 μm, 5–20 μm or 3–20 μm, 20–180 μm and 180–2,000 μm) as template. For unravelling prokaryotic biodiversity, V4 and V5 hypervariable loops of 16S rRNA genes were co-amplified from the same DNA templates used for 18S barcoding and from DNA obtained from prokaryote-enriched fractions (0.2–1.6 μm and 0.2–3 μm).

Both these barcodes present a combination of advantages: (i) they are universally conserved in length and simple in secondary structure, thus allowing relatively unbiased PCR amplification across eukaryotic and prokaryotic lineages followed by Illumina sequencing, (ii) they include both stable and highly variable nucleotide positions over evolutionary time frames, allowing discrimination of taxa over a significant phylogenetic depth, (iii) they are extensively represented in public reference databases across the eukaryotic and prokaryotic tree of life, allowing taxonomic assignment amongst all known lineages.

*3.1. Eukaryotic 18S rRNA gene amplicon generation (Method ID: 18S_PCR)*

For generation of 18S barcodes, PCR amplifications were performed with the Phusion High Fidelity PCR Master Mix with GC buffer (ThermoFisher Scientific) and the forward/reverse primer pair 1389F 5′- TTGTACACACCGCCC-3′ and 1510R 5′- CCTTCYGCAGGTTCACCTAC-3′^42^. The PCR mixtures (25 μl final volume) contained 5 to 10 ng of total DNA template with 0.35 μM final concentration of each primer, 3% of DMSO and 1X Phusion Master Mix. PCR amplifications (98 °C for 30 s; 25 cycles of 10 s at 98 °C, 30 s at 57 °C, 30 s at 72 °C; and 72 °C for 10 min) of all samples were carried out with a reduced number of cycles to avoid the formation of chimeras during the plateau phase of the reaction, and in triplicate in order to smooth the intra-sample variance while obtaining sufficient amounts of amplicons for Illumina sequencing. PCR products were purified by a modified 0.6x AMPure XP beads (Beckmann Coulter Genomics) cleanup in which the supernatant containing larger DNA fragments was kept and purified with the NucleoSpin Gel and PCR Clean-up kit (Macherey-Nagel). Then, aliquots of purified amplicons were run on an Agilent Bioanalyzer using the DNA High Sensitivity LabChip kit to check their lengths and quantified with a Qubit Fluorometer.

*3.2. Prokaryotic 16S rRNA gene amplicon generation (Method ID: 16S_PCR)*

Prokaryotic barcodes were generated using 515F-Y (5′- GTGYCAGCMGCCGCGGTAA-3′) and 926R (5′- CCGYCAATTYMTTTRAGTTT-3′) 16S primers described by Parada *et al.*^43^. This primer pair encompasses the V4 and V5 hypervariable regions, yielding a product of 400 bp. Triplicate PCR mixtures were prepared as described above for 18S amplification, whereas cycling conditions included a 30 s denaturation step followed by 37 cycles of 98 °C for 10 s, 53 °C for 30 s, 72 °C for 30 s, and a final extension of 72 °C for 10 min. After PCR products cleanup using 0.8x volumes AMPure XP beads, amplicons length and amount were checked as described above.

At the time of publication of this paper, generation of 16S rRNA genes amplicon is still under progress. In Metadata Record, an example of datasets produced by this strategy and available at ENA can be found.

### [4] Sequencing library preparation

All library preparations were performed at Genoscope.

*4.1. Metagenomic library preparation from size fractionated filters DNA (Method ID: MetaG)*When the *Tara*

Oceans project started in 2009, Illumina offered a high throughput system that could enable to gain biological insights for complex samples. The counterpart was to obtain maximum read lengths of 100 bp. As short read lengths may be challenging for *de novo* assemblies, library preparation protocols for complex metagenomics samples were improved in order to generate much longer reads by overlapping and merging read pairs before assembly. For this purpose, a size selection step was added at the end of library preparation obtaining narrowly sized libraries around 300 bp. This corresponded to an insert fragment at around 180 bp, allowing ~20 bp paired read overlaps.

Depending on extracted DNA yields, libraries were prepared manually or in a semi-automatic manner. Genomic DNA was first sheared to a mean target size of 300 bp using a Covaris E210 instrument (Covaris, Woburn, MA). DNA inputs in fragmentation step were 30–100 ng in the case of a downstream manual preparation, or 250 ng for semi-automatized protocol, more demanding in DNA quantity. Size profiles of sheared materials were visualized on an Agilent Bioanalyzer DNA High Sensitivity chip.

In the manual protocol, the resulting fragmented DNA was end-repaired, A-tailed at the 3’end, and ligated to Illumina compatible adapters using the NEBNext DNA Sample Prep Master Mix Set 1 (New England Biolabs). Ligation products were subsequently cleaned up using 1x AMPure XP beads.

For >250 ng input gDNA, end repair, A-tailing, adaptors ligation and a 200–400 bp size selection were performed using the SPRIWorks Library Preparation System and SPRI TE instrument (Beckmann Coulter Genomics), according to the manufacturer protocol. This allowed to process rapidly and with few hands-on time, up to 10 samples in parallel.

Ligation products were then enriched by performing 12 cycles of amplification (98 °C for 30 s, 12 cycles of 10 s at 98 °C, 30 s at 60 °C, 30 s at 72 °C and 72 °C for 5 min) using Platinum Pfx Taq Polymerase (Thermo Fisher Scientific) and P5 and P7 primers. Amplified products were purified using AMPure XP beads (1 volume) and samples were run on a 3% agarose gel in order to size-select gel slices around 300 bp. The excised band (280–310 bp) was finally purified using the Nucleospin Extract II DNA purification kit (Macherey-Nagel).

Later on, further optimizations to the original manual protocol were applied to the processing of samples collected during the *Tara* Oceans Polar Circle campaign (stations 155–210). In particular, after gDNA shearing, libraries were prepared using the NEBNext DNA Sample Prep Master Mix kit with a ‘on beads’ protocol that achieves higher library yields. Performing each reaction step on the same AMPure XP beads used for first purification after end repair minimizes sample losses during the successive clean up steps. Ligation was performed with adapted concentrations of Nextflex DNA barcodes (Bioo Scientific, Austin, TX,) and cleaned up by two rounds of AMPure XP beads purifications.

For higher sample inputs (250 ng), library preparation benefitted of high throughput automatized instruments. End-repair, A-tailing and ligation were made by a liquid handler, the Biomek FX Laboratory Automation Workstation (Beckmann Coulter Genomics), able to perform up to 96 reactions in parallel in half a day. Library amplification was performed using Kapa Hifi HotStart NGS library Amplification kit (Kapa Biosystems, Wilmington, MA) (98 °C for 45 s, 12 cycles of 15 s at 98 °C, 30 s at 60 °C, 30 s at 72 °C and 72 °C for 1 min) instead of Platinum Pfx Taq Polymerase. Amplified library was purified and size-selected as described above.

*4.2. Library preparation from viral samples (Method ID: MetaG_virus)*

Due to very low DNA extraction yields obtained from concentrated viral samples (usually only a few nanograms), library preparation protocol was adapted in order to improve its efficiency starting from very low input DNA. Following an extensive study of the impact of DNA amount, amplification and library preparation protocol^36^, the method developed at Genoscope and described in detail at https://www.protocols.io/groups/sullivan-lab was chosen for preparation of all viral metagenomics libraries from *Tara* Oceans stations.

Briefly, 10–15 ng DNA were fragmented to a 150–600 bp size range using the E210 Covaris instrument. End repair, A-tailing and ligation with adjusted concentrations of homemade adaptors were performed using the NEBNext DNA Sample Preparation Reagent Set 1 (New England Biolabs). After two consecutive 1x AMPure XP clean ups, the ligated product was amplified by 12 cycles PCR using Platinium Pfx DNA polymerase followed by 0.6x AMPure XP purification.

For samples collected during the *Tara* Oceans Polar Circle campaign, similar optimizations were applied as described for metagenomics libraries. Manual ‘on beads’ protocol was used on lower inputs (1.3–20 ng). The ligated product was amplified by 12 to 18 cycles PCR using Kapa Hifi HotStart NGS library Amplification kit and purified by 0.6x AMPure XP clean up.

*4.3. Library preparation from SAGs (Method ID: MetaG_SAGs)*

A fixed volume (7.5 μl of single cell-amplified DNA) was used as input for DNA shearing. Then, the same library preparation protocol used for viral libraries was applied without significant modifications.

*4.4. Metatranscriptomic libraries*

Different cDNA synthesis protocols were applied according to the fractions from which RNA originated. A first problem to be solved was to limit the generation of rRNA reads coming from this predominant RNA fraction. In the case of RNA issued from fractions enriched in protists and metazoans (0.8–5 μm (or 0.8–3 μm), 0.8–2,000 μm, 3–2,000 μm, 5–20 μm (or 3–20 μm), 20–180 μm and 180–2,000 μm membrane filters), methods including a poly(A)^+^ RNA selection step were chosen. Whereas this approach is very efficient in lowering the number of rRNA reads, it does not allow to retrotranscribe mRNAs from prokaryotic species, thus leading to eukaryote-only metatranscriptomes.

In contrast, cDNA synthesis from prokaryote- and virus-enriched fractions RNAs (0.2–1.6 μm and 0.2–3 μm) was performed by a random priming approach, preceded by a prokaryotic rRNA depletion step. This method allows cDNA synthesis from both eukaryotic and prokaryotic mRNA and organellar transcripts but also from residual, poorly-depleted eukaryotic rRNA resulting in high percentage of rRNA reads when small protists are abundant.

#### cDNA synthesis and library preparation from eukaryote-enriched fractions

The quantity of extracted total RNA was an additional factor, which conditioned the choice of cDNA synthesis method. When at least 2 μg total RNA were available, cDNA synthesis was carried out using the TruSeq mRNA Sample preparation kit (Illumina, San Diego, CA) (Method ID: TS_RNA). Briefly, poly(A)^+^ RNA was selected with oligo(dT) beads, chemically fragmented and converted into single-stranded cDNA using random hexamer priming. Then, the second strand was generated to create double-stranded cDNA. Next, library preparation was performed according to the protocol described for viral metagenomics libraries by omitting cDNA shearing and performing a post-PCR 1x AMPure XP purification.

In 2012, Illumina released a new version of the kit, the TruSeq Stranded mRNA kit, which allows retaining strand information of RNA transcripts (sequence reads occur in the same orientation as anti-sense RNA). Strand specificity is achieved by quenching the second strand during final amplification thanks to incorporation of dUTP instead of dTTP during second strand synthesis. As strand orientation provides additional valuable information for downstream RNAseq data analysis, this method (Method ID: TS_strand) was applied for processing RNA from samples collected during the Polar Circle campaign. The minimal RNA input used for this library was 1 μg total RNA. After second strand synthesis, ready-to-sequence Illumina library was generated following the manufacturer’s instructions using the reagents included in the kit.

RNA extractions yielding insufficient quantities for TruSeq preparations were processed using the SMARTer Ultra Low RNA Kit (Clontech, Mountain View, CA) (Method ID: SMART_dT). This method, successfully used for eukaryotic single cell transcriptomic studies^44,45^, converts poly(A)^+^ RNA to full-length cDNA using a modified oligo(dT) primer combined with SMART (*S*witching *M*echanism *a*t the 5′ end of *R*NA *T*emplate) technology. Fifty nanograms or less total RNA were used for cDNA synthesis, followed by 12 cycles of PCR preamplification of cDNA. Before Illumina library preparation, 5–50 ng double stranded cDNA were fragmented to a 150–600 bp size range using the E210 Covaris instrument. Then, sheared cDNA were used for Illumina library preparation following the protocol described for viral metagenomes libraries, except for the post amplification AMPure XP purification performed at a ratio 1:1.

#### cDNA synthesis and library preparation from prokaryote- and virus-enriched fractions

As for eukaryotic RNA, the extraction yields from 0.2–1.6 μm and 0.2–3 μm filters were a concern and motivated a preliminary study of different ‘low input’ cDNA synthesis methods adapted to prokaryotic mRNA^46^. On the basis of the results presented in this paper, we chose to perform bacterial rRNA depletion followed by cDNA synthesis with SMARTer Stranded RNA-Seq Kit (Clontech). This method is a more recent release from Clontech than the SMARTer Low Input library kit. Differently from this oligo(dT)-based method, the SMARTer Stranded kit is based on initial chemical RNA fragmentation followed by a first cDNA strand synthesis by random priming and SMART template switching technology. Then, single-stranded cDNA is directly amplified with oligonucleotides which contain Illumina adaptors and indexes sequences to obtain a ready-to-sequence library. Finally, differently from oligo(dT) method, this one preserves the coding strand information which can be deduced after paired end sequencing of library fragments.

Bacterial rRNA depletion was carried out using Ribo-Zero Magnetic Kit for Bacteria (Epicentre Biotechnologies). Different total RNA inputs were depleted, varying between undetectable quantities by Qubit measurement up to 4 μg. Therefore, Ribo-Zero depletion protocol was modified to be adapted to low RNA input amounts according to Alberti *et al.*^46^. Except for these modifications, depletion was performed according to the manufacturer instructions. Depleted RNA were concentrated to 10 μl total volume with RNA Clean and Concentrator-5 kit (ZymoResearch) following the procedure described for retention of >17 nt RNA fragments. Then, when total RNA input was > or equal to 1 μg, depleted RNA amount was checked by Qubit RNA HS Assay quantification and 40 ng, or less, were used to synthetize cDNA with SMARTer Stranded RNA-Seq Kit (Method ID: RiboZero_SMART_Strand). Otherwise, 7 μl were used for cDNA synthesis. Single stranded cDNA was purified by two rounds of purification with 1x AMPure XP beads. The purified product was amplified by 18 cycles PCR with SeqAmp DNA polymerase and the Illumina Index Primer set, both provided in the kit. Final library was purified with 1x AMPure XP beads.

*4.5. Library preparation from V9-18S rRNA amplicons (Method ID: MetaBar_18S)*

In order to evaluate the eukaryotic biodiversity of samples, libraries were prepared from amplicons generated by the amplification of the V9 region of the 18S rRNA gene. As the amplicon size, visualized on an Agilent Bioanalyzer, was around 160 bp (majority peak), no fragmentation was needed before library preparation. Amplicons (100 ng) generated from *Tara* Oceans samples were end-repaired, A-tailed and ligated with Illumina adaptors using the SPRIWorks Library Preparation System and SPRI TE instrument, without any size selection. Ligated products were amplified using Platinum *Pfx* Taq Polymerase and cleaned up on magnetic beads as described above for metagenomic libraries except that gel size selection was skipped.

*Tara* Oceans Polar Circle amplicons were treated as described in metagenomic libraries section for samples issued from the same campaign.

*4.6. Library preparation from V4-V516S rRNA amplicons (Method ID: MetaBar_16S)*

Tags generated from amplification of V4 and V5 hypervariable regions of 16S rRNA genes were used for preparation of sequencing libraries by high throughput automatized instruments. One hundred ng amplicons were directly end-repaired, A-tailed and ligated to Illumina adapters on a Biomek FX Laboratory Automation Workstation. Then, library amplification was performed using Kapa Hifi HotStart NGS library Amplification kit with the same cycling conditions applied for metagenomics libraries. After AMPure XP purification (1 volume) and quantification by Qubit fluorometric measurement (HS assay), equimolar pools of amplified products were run on a 2% agarose gel to select 500–650 bp gel slices (amplicon size increased by Illumina adapters). This sizing step allowed isolating the prokaryotic 16S amplicon from non-specific amplification products. The library was finally purified using the Nucleospin Extract II DNA purification kit.

### [5] Sequencing and data quality control

*5.1. Sequencing library quality control*

All libraries were quantified first by Qubit dsDNA HS Assay measurement and then by qPCR with the KAPA Library Quantification Kit for Illumina Libraries (Kapa Biosystems) on an MXPro instrument (Agilent Technologies). Library profiles were assessed using the DNA High Sensitivity LabChip kit on an Agilent Bioanalyzer. Later on, the quality control step was implemented with quantification by PicoGreen method on 96-well plates and high throughput microfluidic capillary electrophoresis system for library profile analysis (LabChip GX, Perkin Elmer, Waltham, MA).

*5.2. Sequencing procedures*

Libraries concentrations were normalized to 10 nM by addition of Tris-Cl 10 mM, pH 8.5 and then applied to cluster generation according to the Illumina Cbot User Guide (Part # 15006165). Libraries were sequenced on Genome Analyzer IIx, HiSeq2000 or HiSeq2500 instruments (Illumina) in a paired-end mode. Read lengths were chosen in order to produce data fitting with bioinformatics analyses needs (Table 2).

Metabarcoding and metatranscriptomic libraries were characterized by low diversity sequences at the beginning of the reads related respectively to the presence of primer sequence used to amplify 18S and 16S tags and low complexity polynucleotides added during cDNA synthesis. Low-diversity libraries can interfere in correct cluster identification, resulting in drastic loss of data output. Therefore, loading concentrations of these libraries (8–9 pM instead of 12–14 pM for standard libraries) and PhiX DNA spike-in (10% instead of 1%) were adapted in order to minimize the impacts on the run quality.

Sequencing was performed according to the Genome Analyzer IIx User Guide (Part # 15018814), HiSeq2000 System User Guide (Part # 15011190) and HiSeq2500 System User Guide (Part # 15035786).

*5.3. Data quality control and filtering*

**Figure 2:**
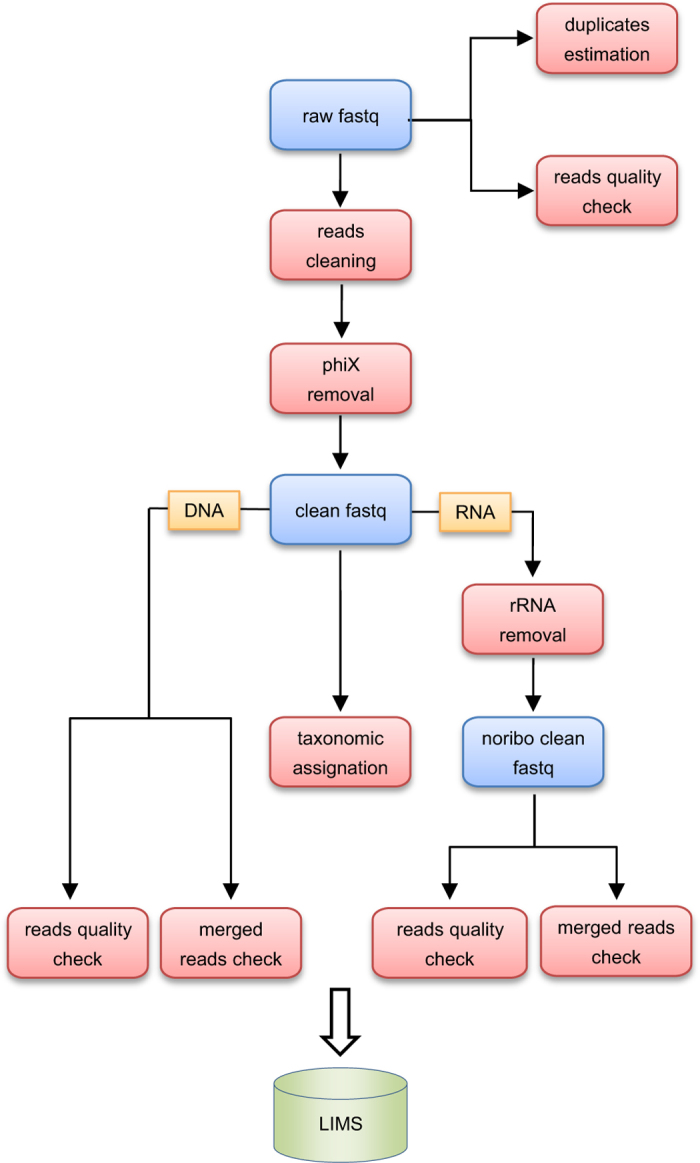
Data processing flowchart.

A first step in data quality control process was the primary analysis performed during the sequencing run by Illumina Real Time Analysis (RTA) software (Code availability 1). This tool analyses images and clusters intensities and filters them to remove low quality data. Furthermore, it performs basecalling and calculates Phred quality score (Q score), which indicates the probability that a given base is called incorrectly. Q score is the most common metric used to assess the accuracy of the sequencing experiment (http://www.illumina.com/documents/products/technotes/technote_Q-Scores.pdf). After conversion of raw BCL files generated by RTA to fastq demultiplexed data by Illumina bcl2fastq Conversion software (Code availability 2), in-house filtering and quality control treatments developed in Genoscope were applied to reads that passed the Illumina quality filters (named raw reads). The parameters of these controls are indicated in Fig. 2.

This processing allows obtaining high quality data and improves subsequent analyses.

Filtering steps were applied on whole raw reads as follows:

- The sequences of the Illumina adapters and primers used during library construction were removed from the whole reads. Low quality nucleotides with quality value<20 were removed from both ends. The longest sequence without adapters and low quality bases was kept. Sequences between the second unknown nucleotide (N) and the end of the read were also trimmed. Reads shorter than 30 nucleotides after trimming were discarded. These trimming steps were achieved using fastx_clean (Code availability 3), an internal software based on the FASTX library (Code availability 4).
- The reads and their mates that mapped onto run quality control sequences (Enterobacteria phage PhiX174 genome, Data Citation 1) were removed using SOAP aligner^47^.
- A specific filter aiming to remove ribosomal reads was applied to data generated from metatranscriptomic libraries sequencing: briefly, the reads and their mates that mapped onto a ribosomal sequences database were filtered using SortMeRNA v 1.0 (ref. 48), a biological sequence analysis tool for filtering, mapping and OTU-picking NGS reads. It contains different rRNA databases and we used it to split the data into two files: rRNA reads in a file (ribo_clean) and other reads in another file (noribo_clean).

Data quality control was performed on random subsets of 20,000 reads before (raw reads) and/or after filtering steps (clean reads) as follows:

- Duplicated sequences rates were estimated from single and paired sequences on raw reads, using fastx_estimate_duplicate (Code availability 5), an internal software based on the FASTX library.
- Read size, quality values, N positions, base composition were calculated and known adapters sequences were detected before and after filtering the reads.
- Taxonomic assignation was performed by aligning with Mega BLAST (Blast 2.2.15 suite)^49^ the subset of 20,000 reads against the nt database (http://www.ncbi.nlm.nih.gov/nucleotide), and using Megan software (version 3.9)^50^.
- The merging step was done with fastx_mergepairs (Code availability 6), an internal software based on the fastx library. The first 36 nucleotides of read2 were extracted and alignment performed between that seed and read1. Merging was launched if the alignment was at least of 15 nucleotides, with less than 4 mismatches and an identity percent of at least 90%. For each overlapping position, the nucleotide of higher quality was retained.

Each dataset was evaluated using specific toolboxes generated from this pipeline (see Technical validation paragraph).

### Code availability

1. Real Time Analysis software: http://support.illumina.com/sequencing/sequencing_software/real-time_analysis_rta/downloads.html
2. Bcl2fastq Conversion: http://support.illumina.com/sequencing/sequencing_software/bcl2fastq-conversion-software.html
3. Fastx_clean software, http://www.genoscope.cns.fr/fastxtend
4. *FASTX-Toolkit*, http://hannonlab.cshl.edu/fastx_toolkit/index.html
5. fastx_estimate_duplicate software, http://www.genoscope.cns.fr/fastxtend
6. fastx_mergepairs software, http://www.genoscope.cns.fr/fastxtend

## Data Records

This data descriptor provides an opportunity to present collections of different datasets generated from sequencing analysis of samples collected during *Tara* Oceans expedition. Fastq files produced from sequencing experiments are available in the global repository for public nucleotide sequence data, the International Nucleotide Sequence Database Collaboration (INSDC, http://www.insdc.org/about) under the umbrella project permanent identifier PRJEB402 ‘Tara-oceans samples barcoding and shotgun sequencing’ (Data Citation 2). Nucleotide sequence information has been deposited via the European gateway to the INSDC, the European Nucleotide Archive (ENA, http://www.ebi.ac.uk/ena) at the EMBL European Bioinformatics Institute (EMBL-EBI). Nucleotide sequence data of each sequencing strategy applied to the size-fractioned *Tara* Oceans plankton communities are registered in a separate component project and linked to the PRJEB402 umbrella project. For instance, the metatranscriptome sequencing of samples from the size fraction of protists is registered under the component project with the identifier PRJEB6609 and available at http://www.ebi.ac.uk/ena/data/view/PRJEB6609. Each generated nucleotide sequence file is associated with a sample record containing extremely rich information on the environment of the corresponding sequenced *Tara* Oceans sample. The sample contextual data available with nucleotide sequences map to the environmental and biogeochemical measurements for each *Tara* Oceans sample available in the Sample Registry at PANGAEA (Data Citation 3). A list of available FASTQ files with repository information is presented in the associated Metadata Record and is also available at PANGAEA (Data Citation 4). Most importantly, this metadata document allows to link each FASTQ file to the experimental protocols used for their generation.

In order to help scientists to correctly manipulate these data, it is important to underline that in many cases, two (or, sporadically, more) FASTQ files were generated by repeated sequencing of the same library. Consequently, FASTQ files sharing the same library name should be pooled for bioinformatics analyses.

This high contextualization of all *Tara* Oceans sequence data makes the whole dataset a unique and valuable tool to marine ecosystem biologists and can serve as an example to other large-scale data generating projects.

## Technical Validation

### Sample and experiments information management

All samples received by Genoscope and all experiments performed from nucleic acid extractions to sequences generation were tracked by an in-house Laboratory Information Management System (LIMS). This LIMS is designed to accumulate information on each sample at each step of the processes. This software has been essential for internal follow-up at any stage of the processing of such a huge amount of samples. With this approach, all collecting data (station, depth, porosity), experiment data (protocol and quality control results) and bioinformatics quality control analyses (duplicates, contamination, taxonomy, mapping, merging) follow each sample throughout the experiment chain until sequencing data analysis. All these properties can be used to search and display sample data in reports during all the process.

### Quality control during sample processing

**Figure 3:**
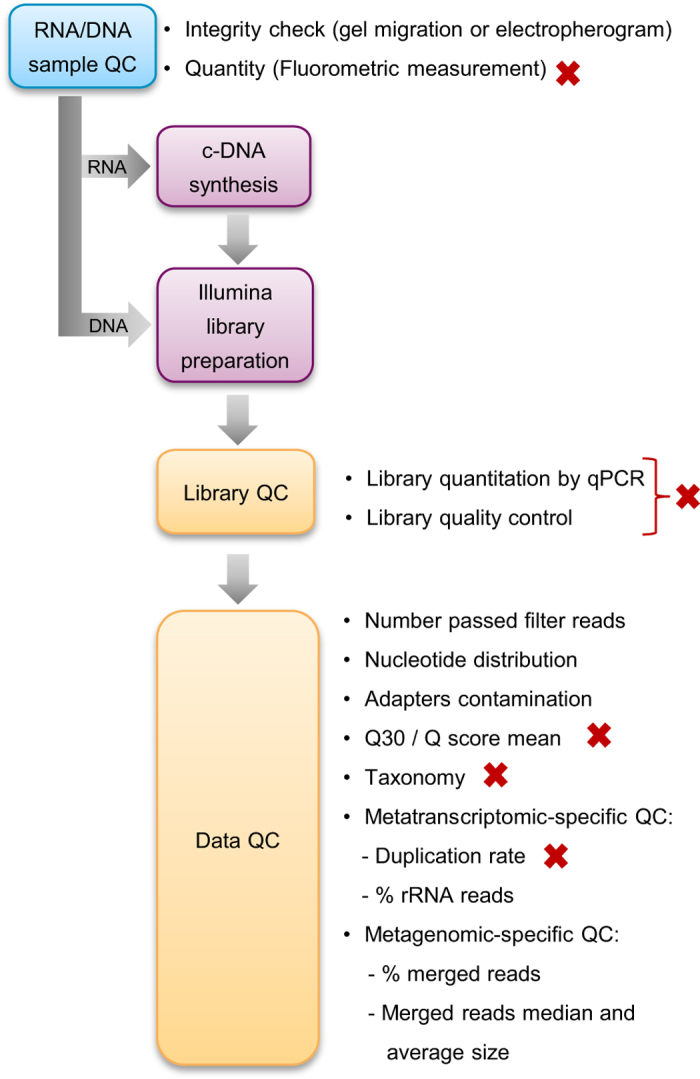
Overview of experimental pipeline from nucleic acids to sequences. Red crosses highlight QC steps where experiments can be stopped.

The pipeline for complete conversion of nucleic acids into sequences included various check points at which sample processing was stopped if the experiment did not meet some well-defined quality criteria (Fig. 3).

*Quality control: DNA and RNA integrity*Integrity of DNA extracted from size-fractionated filters was checked by running a DNA aliquot from a subset of samples on 0.7% agarose gel. DNA quality was visually evaluated by comparing migration to high molecular weight markers. Usually, the majority of the DNA should be located on a tight band at high molecular weight. However, a smear was present in the majority of the samples, indicating partial DNA degradation.

RNA quality was evaluated by capillary electrophoresis migration on an Agilent Bioanalyzer, using the RNA 6,000 Pico LabChip kit. Total Eukaryotic RNA Assay was selected for electropherogram internal analysis for RNA extracted from protist- and metazoan-enriched filters whereas Total Prokaryotic RNA Assay was applied to prokaryote-enriched filters. These software allow generation of a RNA Integrity Number (RIN) calculated by comparing rRNA peaks with a specific database (eukaryotic or prokaryotic or plants) and usually used as a score of RNA quality. In many *Tara* Oceans RNA samples, particularly from 0.8–5 μm, 0.22–1.6 μm and 0.22–3 μm filters, eukaryotic and prokaryotic species were co-extracted, generating atypical rRNA peaks profiles. For this reason, the RIN was not accurate or even not computable and did not reflect the quality of the preparations. However, as for DNA preparations, RNA quality was sometimes poor as most Agilent profiles showed rRNA peaks but also variable amounts of small size RNA, indicating partial degradation.

DNA and RNA low qualities probably reflected the difficulty to preserve the integrity of very complex and heterogeneous communities all along the different steps from sampling on board to extraction in the lab. The particular origin and natural variability of the sampled biomass had an impact also on nucleic acid extractions yields, evaluated by Qubit quantitation. These were highly variable and usually reflected the abundance of collected plankton at a given sampling point. Samples under the minimal required amount for each specific library preparation (as indicated in the Methods section) were not further processed.

#### Quality control: Sequencing library

**Figure 4:**
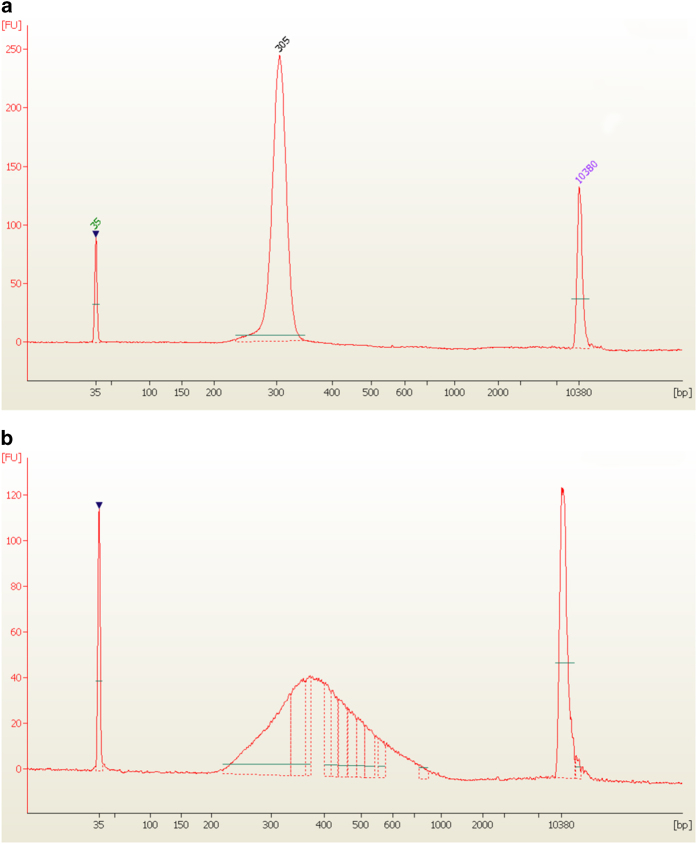
Agilent Bioanalyzer profiles of amplified libraries. (**a**) Shows an example of electropherogram obtained following the metagenomic library preparation protocol described in paragraph 4.1. The size of this kind of library is very tight due to the size selection step for generation of overlapping paired end reads. (**b**) Shows an example of metatranscriptomic library generated following the TS_RNA protocol.

The qualitative and quantitative analyses performed on the ready-to-sequence libraries were a crucial step for achieving high quality sequences. First, library size profiles obtained on Agilent or LabChip instruments traces were carefully evaluated and validated only if they corresponded to what expected from the specifically applied library construction protocol. As an example, metagenomics libraries preparations which included a tight gel size selection step for generation of overlapping reads (see paragraph 4.1), should generate a discrete peak at around 300 bp corresponding to the insert size (~180 bp) increased by the addition of Illumina adapters (120 bp) (Fig. 4a). In contrast, a standard TruSeq metatranscriptomic library (section 4.4) should cover a broader size range between 200 and ~600 bp (Fig. 4b).

Even if at the end of preparation, libraries were immediately quantified by a Qubit measurement, a qPCR quantitation was systematically performed as recommended by Illumina company and the obtained value was retained for library normalization to 10 nM. Indeed, previous experience showed that qPCR is much more accurate in order to create optimum cluster densities across every lane of the flow cell.

*Quality control: Data validation*For the *Tara* Oceans project, specific report configurations were developed within internal LIMS to display, compare and analyse hundreds of samples.

**Figure 5:**
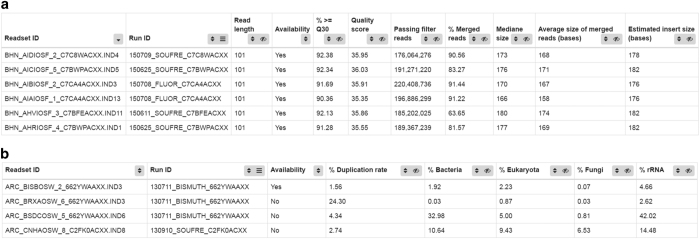
Representative examples of tabulated data reports generated by the LIMS for multiple datasets. (**a**) Shows an example of sequencing report for metagenomics libraries. Metrics particularly useful for evaluating the quality of this type of data can be visualized, as the % of merged reads, the median size length and the estimated insert size. (**b**) Shows an example of report for metatranscriptomic libraries from poly(A)^+^ RNA. Quality control of these libraries focuses on duplication rate and potential contamination by bacteria and fungi, whose % are easily visualized on the report.

**Figure 6:**
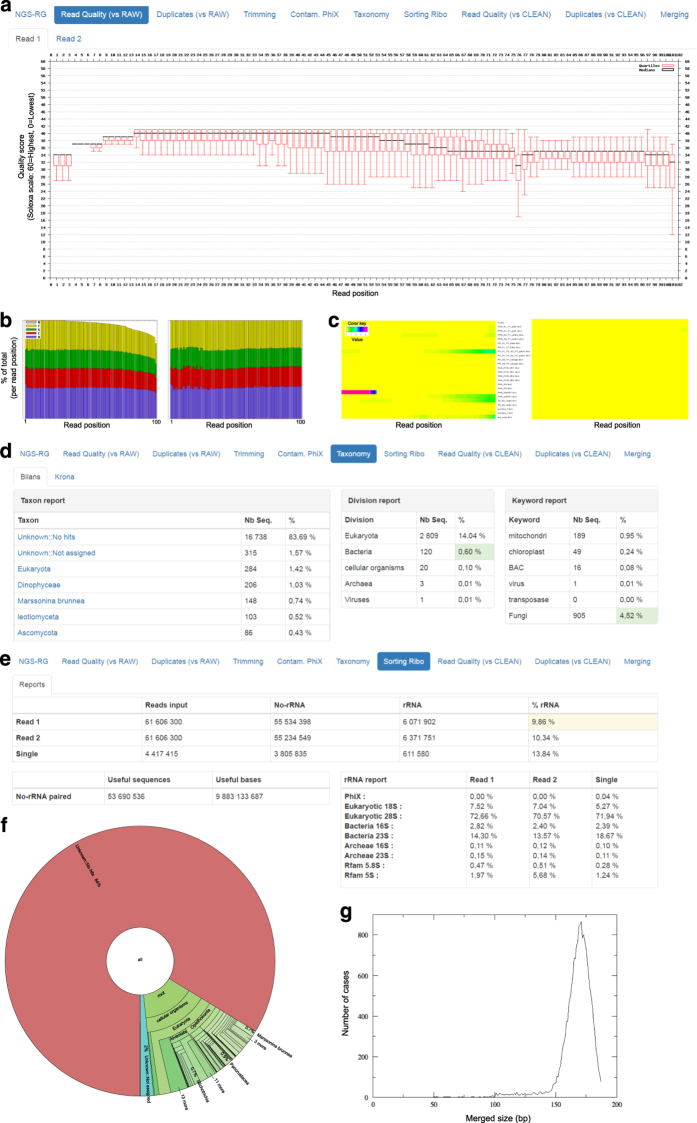
Representative examples of key data reports generated by the LIMS for individual datasets. (**a**) Quality score box plot of 100-bp Illumina reads. This plot summarizes the average quality per position over all reads; it shows the box-plot per position in the read and the average smoothed line in black. (**b**) Nucleotide distribution chart per read position: at left, before adapters and low quality reads trimming; at right, after the trimming process. On the left plot, a non-random distribution in the first 12 bases is typical of metatranscriptomic libraires generated with SMART-dT protocol, which leaves SMARTer adapter sequencing at the beginning of the cDNA insert. (**c**) Graphical representation of known overrepresented sequences (primers and adapters used for library preparation) before (left panel) and after (right panel) adapter sequences trimming. Again, the overrepresentation of SMARTer adapter is easily visualised on the left panel (red bar) and it disappears after the trimming process (right panel). (**d**) Report of taxonomic assignation by organism (left), by division (middle) and by keyword (right). Bacteria and fungi %<5% are highlighted in green to facilitate manual validation of the dataset. (**e**) Report of rRNA sequences detection and trimming with detail of % of different rRNA species. (**f**) Krona chart of the same taxonomic assignment reported in (**d**). (**g**) Distribution of the length of the reads obtained after merging of paired reads generated by sequencing of a metagenomic library.

The quality of each dataset was assessed using the interfaces depicted in Figs 5 and 6 which provided a quick insight into the dataset quality allowing smart check of important statistics as the number of passed filter reads and duplication rate; % rRNA reads for metatranscriptomic datasets; and % merged reads, their median and average size for metagenomics ones. Furthermore, for each sample, plots and graphics were generated and allowed to easily visualize base quality (Q score) and nucleotide distribution before and after the filtering treatments described in paragraph 5.3, as well as taxonomical assignments (Fig. 6).

Among all the sequences quality control statistics, the following parameters were considered crucial for identification of low quality sequences and drove decision about passing quality control:

- Base calling accuracy measured by Phred quality score (Q score): for each sample, this metric was illustrated by the calculation of Q score mean and % of bases with Q≥30 (Fig. 5a) as well as a quality score plot (Fig. 6a). A Q score of 30 means the probability of an incorrect base call 1 in 1,000 times, in other words that the base call accuracy is 99.9%. Generally, datasets were valid when Q score mean was >30 and % of bases with Q≥30 was at least 80%. However, this last criterion was not applied to metatranscriptomics and amplicon libraries as the particular library construction and the low base diversity, especially in the case of amplicons, had a negative impact on the Q score by decreasing it significantly.
- Taxonomic assignation (Fig. 6d,f): the majority of the reads were classified under the ‘no hits’ or ‘not assigned’ item, as expected from the origin of the samples. Otherwise, the sequences were assigned to known marine species. The presence of other species not expected to live in ocean environment was considered as contaminating the sample and a threshold of 2% per species was arbitrarily defined to invalidate the dataset. However, this kind of contamination was rare and in most cases attributed to *Homo sapiens* DNA.

Whereas these criteria were applied to all samples, others were defined only for metatranscriptomics samples issued from protists/metazoan-enriched filters. In particular:

- High duplicate rates (calculated on raw paired end reads) were considered as characteristic of low complexity samples. Datasets containing>20% duplicates were not further processed (Fig. 5b).
- Taxonomic assignations attributed to bacteria were not expected in these samples, produced by poly(A)^+^ RNA selection, and considered as a background signal. An arbitrary threshold defined at 5% was applied for passing quality control (Figs 5b and 6d).
- Datasets containing more than 5% reads assigned to the Fungi kingdom were further inspected. Most of them were classified as filamentous ascomycete fungi, a group including both marine and terrestrial species. Verification about the habitat of the suspected contaminant was made before taking decision of discarding the dataset (Figs 5b and 6d).

### Data Availability

The authors declare that all data reported herein are fully and freely available from the date of publication, with no restrictions, and that all of the samples, analyses, publications, and ownership of data are free from legal entanglement or restriction of any sort by the various nations whose waters the Tara Oceans expedition sampled in.

## Additional Information

**How to cite this article:** Alberti, A. *et al.* Viral to metazoan marine plankton nucleotide sequences from the *Tara* Oceans expedition. *Sci. Data* 4:170093 doi: 10.1038/sdata.2017.93 (2017).

**Publisher’s note:** Springer Nature remains neutral with regard to jurisdictional claims in published maps and institutional affiliations.

## Supplementary Material



## Figures and Tables

**Table 1:** Summary of libraries generated from *Tara* Oceans DNA and RNA samples and sequencing experiments performed on each type of library.

**Size fractions (μm)**	**Mainly targeted organisms**	**Targeted genomic analysis**	**Sample storage laboratory**	**Sequencing laboratory**	**Method ID Nucleic acids preparation (Section)**	**Method ID Amplicons generation (Section)**	**Method ID Library preparation (Section)**
<0.2 μM	Viruses	Metagenomics	M. Sullivan lab (University of Arizona, AZ, US)	CEA, Genoscope, France	Virus_DNA_ext (2.4)		MetaG_virus (4.2)
0.2–1.6, 0.1–0.2, 0.45–0.8, 0.2–0.45	Giruses	Metagenomics	N. Grimsley lab (CNRS, Banyuls-sur -Mer, France)	CEA, Genoscope, France	Girus_DNA_ext (2.5)		MetaG (4.1)
0.2–1.6, 0.2–3	Viruses, Giruses, Prokaryotes, small Eucaryotes	16S metabarcoding	S.G. Acinas lab (ICM-CSIC, Barcelona, Spain)	CEA, Genoscope, France	Acinas_Prok_DNA_ext (2.2)	16S_PCR (3.2)	MetaBar_16S (4.6)
		Metagenomics			Acinas_Prok_DNA_ext (2.2)		MetaG (4.1)
		Metatranscriptomics by random priming			Acinas_Prok_RNA_ext Genoscope_Prok_RNA_ext (2.2)		RiboZero_SMART_strand (4.4)
0.8-inf, 3-inf, 0.8–5 (0.8–3), 5–20 (3–20), 20–180, 180–2,000	Protists and metazoa	18S metabarcoding	C. De Vargas lab (CNRS/UPMC, Roscoff, France)	CEA, Genoscope, France		18S_PCR (3.1)	MetaBar_18S (4.5)
		16S metabarcoding				16S_PCR (3.2)	MetaBar_16S (4.6)
		Metagenomics	P. Wincker lab (CEA, Genoscope, France)		Euk_ DNA_RNA_ext (2.1)		MetaG (4.1)
		Metatranscriptomics on poly(A)^+^ RNA			Euk_ DNA_RNA_ext (2.1)		TS_RNA (4.4) TS_strand (4.4) SMART_dT (4.4)
Samples for SAGs	Protists	*De novo* sequencing	N. Poulton lab (Bigelow lab, ME, US)	CEA, Genoscope, France	SAGs_amplif (2.6)		MetaG_SAGs (4.3)
Number of libraries with available readsets in public databases at the date of publication of the paper.							

**Table 2:** Summary of libraries generated from *Tara* Oceans DNA and RNA samples and sequencing experiments performed on each type of library.

**Sequencing library preparation method**	**Library insert size (pb)**	**Sequencing instrument**	**Read length (PE mode)**	Generated libraries*	**Mean number of reads per sample (millions of paired reads)**
Metagenomics from size fractionated filters (Section 4.1)	180	HS2000	101	855	160
Metagenomics from viral samples (Section 4.2)	150–900	HS2000	101	90	50
SAGs (Section 4.3)	150–900	HS2000	101	49	20
Metatranscriptomic libraries (Section 4.4)	100–600	HS2000	101	467	160
18S metabarcode libraries (Section 4.5)	160	GAIIx	151	884	1.5
16S metabarcode libraries (Section 4.6)	400	HiSeq2500	251	In progress	ND

*Number of libraries with available readsets in public databases at the date of publication of the paper.
